# From the beginning to the highest impact factor – the journey of World Psychiatry

**DOI:** 10.1192/j.eurpsy.2023.209

**Published:** 2023-07-19

**Authors:** M. Luciano

**Affiliations:** Department of Psychiatry, University of Campania Luigi Vanvitelli, Naples, Italy

## Abstract

**Abstract:**

*World Psychiatry*, the official journal of the World Psychiatric Association, was founded in the year 2002. From the very beginning, its aims have been: a) to reach as many psychiatrists as possible worldwide, disseminating information on recent significant clinical, service and research developments, using a language that can be assimilated by most of them; b) to give voice to psychiatrists of all regions of the world, encouraging submission of research papers, commentaries and reports on innovative service modalities. The two main criteria by which an article submitted to the journal is evaluated have been from the beginning: a) its relevance to everyday clinical practice of the average psychiatrist; and b) its usefulness to foster the average psychiatrist’s professional growth. From its first issue, the journal has been freely available online. In the year 2008, *World Psychiatry* received its first impact factor, that was 3.896. On that same year, the publication of the Spanish edition of the journal was started. The impact factor of *World Psychiatry* has then increased year after year, up to the value of 79.683 reached in July 2022. For the eighth consecutive year, *World Psychiatry* has been no. 1 in the Clarivate’s category of Psychiatry, and for the fifth consecutive year no. 1 in the entire Clarivate’s Social Science Citation Index. All the issues of the journal are now freely available both on the PubMed system and on the WPA website. The journal reaches now, in its online or printed edition, more than 60,000 mental health professionals worldwide. One of the main factors explaining the success of the journal is the continuing attempt to identify in advance topics which are going to become very visible in the international literature and relevant to ordinary clinical practice. The journal has been recently praised by the World Health Organization for the representation of members from low/middle income countries in its Advisory Board, which was at that time 32.3%, while among the other top ten psychiatric journals it was 1.9%. Furthermore, every issue of the journal includes contributions from all continents.

**Disclosure of Interest:**

None Declared

